# Supervised versus unsupervised antimalarial treatment with six-dose artemether-lumefantrine: pharmacokinetic and dosage-related findings from a clinical trial in Uganda

**DOI:** 10.1186/1475-2875-5-59

**Published:** 2006-07-19

**Authors:** Francesco Checchi, Patrice Piola, Carole Fogg, Francis Bajunirwe, Samuel Biraro, Francesco Grandesso, Eugene Ruzagira, Joseph Babigumira, Isaac Kigozi, James Kiguli, Juliet Kyomuhendo, Laurent Ferradini, Walter RJ Taylor, Jean-Paul Guthmann

**Affiliations:** 1 Epicentre, Paris, France; 2 Department of Infectious and Tropical Diseases, London School of Hygiene and Tropical Medicine, London, UK; 3 Mbarara University of Science and Technology, Mbarara, Uganda; 4 UNICEF/UNDP/World Bank/WHO Special Programme for Research and Training in Tropical Diseases, Geneva, Switzerland

## Abstract

**Background:**

A six-dose antimalarial regimen of artemether-lumefantrine (A/L) may soon become one of the most widely used drug combination in Africa, despite possible constraints with adherence and poor absorption due to inadequate nutrition, and a lack of pharmacokinetic and effectiveness data.

**Methods:**

Within a trial of supervised versus unsupervised A/L treatment in a stable Ugandan *Plasmodium falciparum *transmission setting, plasma lumefantrine concentrations were measured in a subset of patients on day 3 (C [lum]_day3_) and day 7 (C [lum]_day7_) post-inclusion. Predictors of lumefantrine concentrations were analysed to show how both C [lum]_day7 _and the weight-adjusted lumefantrine dose affect 28-day recrudescence and re-infection risks. The implications of these novel findings are discussed in terms of the emergence of lumefantrine-resistant strains in Africa.

**Results:**

C [lum]_day3 _and C [lum]_day7 _distributions among 241 supervised and 238 unsupervised patients were positively skewed. Unsupervised treatment and decreasing weight-adjusted lumefantrine dose were negatively associated with C [lum]_day3_. Unsupervised treatment and decreasing age showed strong negative associations with C [lum]_day7_. Both models were poorly predictive (R-squared < 0.25). There were no recrudescences in either arm, but decreasing lumefantrine dose per Kg resulted in up to 13-fold higher adjusted risks of re-infection. Re-infections occurred only among patients with C [lum]_day7 _below 400 ng/mL (p < 0.001).

**Conclusion:**

Maintaining the present six-dose regimen and ensuring high adherence and intake are essential to maximize the public health benefits of this valuable drug combination.

## Background

The fixed dose antimalarial combination of artemether-lumefantrine (A/L) is a promising and efficacious artemisinin-based combination treatment (ACT) that could play a key role in reducing the high mortality suffered by African children with *Plasmodium falciparum *malaria. The three day, six dose regimen of A/L is currently prioritized by the World Health Organization as a replacement for failing antimalarial monotherapies, notably chloroquine and sulfadoxine-pyrimethamine (SP). Several countries (e.g. Zambia, Kenya, Tanzania, South Africa, Niger, and Uganda) have now adopted A/L as the first line antimalarial. Clinical trials have shown A/L to be safe and highly efficacious [1]. However, in routine practice, several drawbacks may reduce its effectiveness. Absorption of the longer-acting, lipophilic partner drug, lumefantrine, is highly dependent on the intake of food, especially lipids. Its oral bioavailability varied sixteen-fold in fasting healthy Thai volunteers compared to volunteers who had taken a fatty meal. Taking a light (500 calories, 10 g fat) or 'normal' (1,000 calories, 20 g fat) meal within one hour either side of the first A/L dose increased lumefantrine bioavailability by a mean factor of 48% and 108%, respectively, compared to fluids alone[2]. In these volunteers, the relative bioavailability increased three-fold with the third and fourth doses compared to the first two doses because appetite improved rapidly in parallel with clinical recovery. There is also considerable inter-individual variation in the peak plasma lumefantrine concentration (C_max_) in volunteers and in patients [2]. In Thai patients, the lower and upper fifth percentile C_max _of the three day, six dose regimen were 1,100 and 19,800 ng/mL respectively, an eighteen-fold difference [3].

The spacing of each dose is also important according to the manufacturer (Novartis Pharma, Basel, Switzerland). The first and second doses should be taken eight hours apart. The remaining doses (two doses on the subsequent two days) should be taken 12 hours apart with the third dose taken 24 hours after the first. To help with this complex regimen, A/L is currently packaged in an illustrated, patient-friendly blister. Assessment of this packaging in Mbarara, Uganda resulted in high rates of adherence and a day 3 median (range) lumefantrine concentration in patients of all ages of 2,450 (600 to 11,400) ng/mL [4]. The median concentration in children under five, however, was significantly lower than in older children and adults [4].

Outside the research setting, poor adherence might compound the problems of poor lipid intake and dose spacing, and further contribute to sub-optimal lumefantrine blood concentrations. Dose ranging studies have established that the key pharmacokinetic (PK) determinant of cure for A/L is the area under the concentration time curve (AUC) of the longer acting lumefantrine, and, therefore, the time during which lumefantrine concentrations exceed both the minimum parasiticidal (MPC) and minimum inhibitory (MIC) concentrations of the parasites. In multi-drug resistant areas of Thailand, the day 7 lumefantrine concentration was a useful surrogate for AUC. A concentration of less than 280 ng/ml was a useful predictor of treatment failure and may approach the *in vivo *MIC of parasites from this area [2,3]. Factors leading to a lowering of the AUC will reduce the probability of cure and could shorten the therapeutic lifespan of A/L, by exposing parasites to sub-therapeutic lumefantrine concentrations, thus favouring the emergence of lumefantrine-resistant strains.

Pharmacokinetic studies of lumefantrine have been conducted in Chinese, Thai and European adult patients and in a very small number of children between 6 and 12 years of age [2,3,5-7], but published data from sub-Saharan Africa, where the drug is most likely to see widespread use, are lacking. In particular, young children under five are conspicuously absent from the literature. Our group previously reported a trial of supervised versus unsupervised A/L for treatment of uncomplicated falciparum malaria in Ugandan patients of all ages. Both methods of treatment administration were highly effective, achieving cure rates exceeding 95% [8]. Here, lumefantrine pharmacokinetic findings from a subset of these patients are presented, and their determinants and effects on novel infections during 28 days of follow up are examined.

## Methods

### Study procedures

Trial methods are detailed elsewhere [8]. Briefly, the study was conducted in Mbarara, southwestern Uganda, an area of multi-drug resistant *P. falciparum *[9,10]. Ethical approval was obtained from Mbarara University of Science and Technology and from the Uganda National Council for Science and Technology. After obtaining written informed consent, non-pregnant, uncomplicated malaria patients with symptomatic *P. falciparum *mono-infection (500 to 100,000/μL) were randomized to a six dose regimen of either supervised or unsupervised A/L (Novartis Pharma, Basel, Switzerland), according to body weight: (i) one tablet (artemether 20 mg/lumefantrine 120 mg) per dose (10–14.9 Kg), (ii) two tablets/dose (15–24.9 Kg), (iii) three tablets/dose (25–34.9 Kg), and (iv) four tablets/dose (≥35 Kg). In the supervised arm, each dose administration was directly observed for 30 minutes, and the whole dose was repeated in case of vomiting. 300 mL milk (~10 g fat) and 30 g groundnuts (~13 g fat) were given with each supervised dose. For small children, the tablet was dissolved in water and spoon-fed, and the fat source consisted of breast milk. Doses one and two were given exactly eight hours apart, dose three was given at 8 a.m. the following morning (about 24 hrs after dose one), and the remaining doses 12 hours apart. Unsupervised patients received the first dose as above, but were given the remainder of the blister pack for home administration, along with a standardized message on dose spacing and appropriate food intake.

Patients were assessed on days 3, 7, 14, 21 and 28 for safety and efficacy endpoints. Recurrent parasitaemias between days 7 and 28 were analysed by a polymerase chain reaction (PCR) comparing the pre- and post-treatment genotypes of the parasite loci coding for the merozoite surface proteins 1 and 2 (MSP-1 and MSP-2) and the glutamate rich protein (GLURP)[11], so as to distinguish new from recrudescent infections. Parasitological failure was defined as any occurrence of PCR-confirmed recrudescent parasitaemia during follow-up (irrespective of symptoms).

Sampling in the trial was stratified to compare cure rates in three age groups: under 5 years, 5–14 years, and 15 and above. In each arm and age group, 70 patients per group were randomly sampled for plasma lumefantrine levels on days 3 and 7 (i.e. 210 supervised and 210 unsupervised). These patients were selected using simple random sampling from the treatment allocation lists. Venous blood (4 mL) was collected in heparinized vacutainers during the days 3 and 7 morning visits. Blood samples were immediately centrifuged and plasma stored in cryotubes at -70°C up to and including transport to Novartis Pharma in Paris, France, where plasma lumefantrine levels were determined blindly using a previously described high performance liquid chromatography (HPLC) method. This method detects C [lum] values > 5 ng/mL, with coefficients of variation of 1.8 to 4.2% [12].

### Statistical analysis

Data were double-entered on EpiData 3.0 (the Epidata Association, Odense, Denmark), and analysed using Stata 8.2 (Stata Corp., College Station, Texas, USA). Lumefantrine concentrations were compared by arm within age groups using the unpaired t-test, and C [lum]_day3 _and C [lum]_day7 _were correlated using Spearman's rho correlation coefficients. The association between baseline patient variables and C [lum]_day3 _or C [lum]_day7 _was then analysed in separate multivariate linear regression models, proceeding as follows. First, the dependent variable (lumefantrine concentration) was transformed into its square root (C [lum]_day3_) or natural logarithm (C [lum]_day7_) so as to normalize distributions. For each patient, the weight-adjusted lumefantrine dose prescribed in mg/Kg was then calculated (= number of tablets per dose × 6 doses × 120 mg/dose/bodyweight in Kg), and categorized as <50, 50–64, 65–79, and ≥80 mg/Kg, which corresponded almost exactly to 1 SD intervals away from the mean of 64 mg/Kg. Certain baseline parameters were selected as independent explanatory variables for inclusion in the models: (i) if they appeared likely to represent plausible biological or epidemiological predictors (referral from hospital outpatient department vs. outlying clinics; gender; history of anorexia, nausea, vomiting, abdominal pain, diarrhoea, or fatigue; hepatomegaly; day 0 axillary temperature; asexual parasite density; haemoglobin; presence of concomitant febrile illness; and co-administration of antibiotics or antihelminthics); or (ii) if they were associated with the outcome at the p < 0.20 level in a univariate analysis (history of dizziness, gametocytaemia). The regression was performed on all selected explanatory variables, and non-significant ones were then progressively removed. Dropped variables were then re-added one by one and retained if they affected the model or were associated significantly with the outcome. Weight-adjusted lumefantrine dose, age group and arm were forced into all final models (this was done since the association of these variables with the outcome seemed very biologically plausible; however, alternative models were also constructed without forcing these variables). After searching for plausible interactions and assessing the effect of influential data points, model assumptions were tested, including normality of residuals, linearity between dependent and explanatory variables, lack of collinearity among explanatory variables, and constant variance (homoscedasticity).

The association between the weight-adjusted lumefantrine dose and the risk of re-infection during the 28 days of follow-up was then analysed by the Cox proportional hazards model, controlling for potential confounders. All enrolled patients who were analysable by intention to treat (ITT) were included in this analysis [8]. Among baseline explanatory variables considered potential confounders (hospital attendance, gender, day 0 axillary temperature, splenomegaly, asexual density, gametocytaemia, and haemoglobin), those with a p < 0.20 in the univariate analysis of the outcome (re-infection) were selected. The final model was constructed manually as described above. After searching for interactions, the proportional hazards assumption that relative risks do not vary with time was tested.

Finally, the Log-Rank test was used to compare the probability of survival (i.e. of remaining re-infection free) from day 7 as a function of the C [lum]_day7 _levels: <200, 200–399, 400–599, and ≥600 ng/mL.

## Results

### Pharmacokinetics

Baseline characteristics of the 479 patients who had at least one pharmacokinetic sampling are shown in Table 1. No C [lum]_day3 _results were available for 22 of these patients (4.6%), and no C [lum]_day7 _results for were available for 24 (5.0%). A further five patients with outlier values were excluded from day 7 analyses. Characteristics of patients with missing data did not significantly differ from those of the analysable sample (data not shown).

**Table 1:** Baseline characteristics of patients sampled for lumefantrine pharmacokinetics.

**Variable**	**Supervised arm**			**Unsupervised arm**		
	< 5 years	5–14 years	≥15 years	< 5 years	5–14 years	≥15 years
		
number sampled	70	92	79	68	91	79
sex ratio (M/F)	1.9 (46/24)	0.9 (43/49)	0.8 (36/43)	1.1 (36/32)	0.7 (37/54)	0.5 (28/51)
median age (range)	3 (1–4)	7 (5–14)	25 (15–60)	3 (1–4)	8 (5–14)	25 (15–80)
parasite density (median, IQR)	12 344 (4232–33 799)	15 668 (3750–36523)	6256 (1957–15 980)	22 777 (11 290–51 775)	11 367 (3449–32 475)	6156 (2749–19 459)
axillary temperature (°C) (mean, IQR)	37.5 (36.7–38.4)	37.3 (36.4–38.0)	36.7 (36.0–37.5)	37.7 (36.7–38.9)	37.1 (36.1–37.8)	36.7 (36.1–37.2)
weight-adjusted lumefantrine dose (mg/Kg) (mean, range)	66 (48–96)	72 (48–96)	53 (32–76)	68 (48–96)	73 (51–96)	54 (35–80)

**Figure 1:**
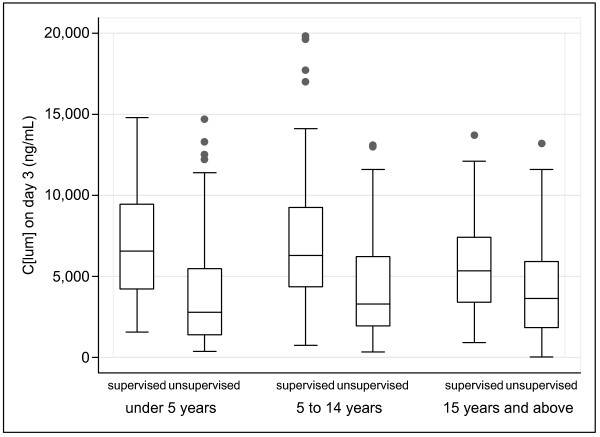
**Box plots of day 3 lumefantrine concentration by age group and arm**. Each box displays the median, upper and lower quartiles of the respective distribution. Box whiskers represent the maximum and minimum range excluding any extreme outliers (shown as dots).

**Figure 2:**
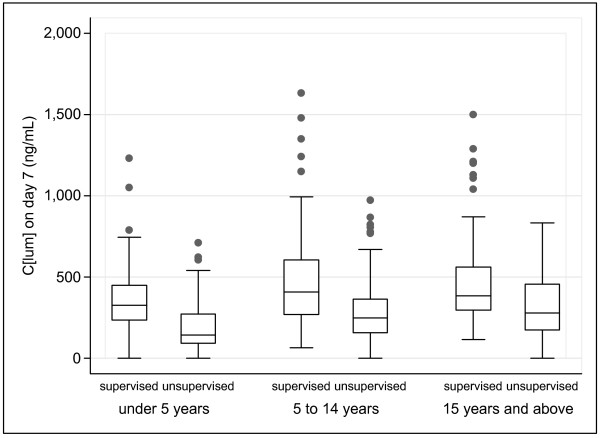
**Box plots of day 7 lumefantrine concentrations by age group and arm**. Each box displays the median, upper and lower quartiles of the respective distribution. Box whiskers represent the maximum and minimum range excluding any extreme outliers (shown as dots).

Lumefantrine concentration distributions were skewed to the right. There was marked inter-individual variability in C [lum]_day3 _and C [lum]_day7 _for all age groups and arms (Figures 1 and 2). Using the fifth and 95^th ^centile concentrations, the variability in each age group (<5, 5–14, ≥15 y) was 13 200/857 (15.4-fold), 12 100/978 (12.4) and 10 700/692 (15.5) respectively for C [lum]_day3 _and 718/58 (12.4), 824/86 (9.6) and 870/101 (8.6) for C [lum]_day7_. Median supervised versus unsupervised C [lum]_day3 _values (ng/mL) were 7040 vs. 2700 (<5 y), 5965 vs. 3310 (5–4 y), and 5320 vs. 3490 (≥15 y) (p < 0.001 for all comparisons). Corresponding median C [lum]_day7 _values were 330 vs. 156, 402 vs. 249, and 382 vs. 281 (p < 0.001 for all comparisons). C [lum]_day3 _and C [lum]_day7 _showed good correlation (Rho = 0.70, p < 0.001), with no differences across age groups or arms. 70/241 (29.0%) supervised and 140/238 (58.8%) unsupervised patients had a C [lum]_day7 _below 280 ng/mL.

In multivariate linear models, unsupervised treatment had a strong negative association with C [lum]_day3 _(Table 2). A history of vomiting was also a negative explanatory variable and a negative trend was apparent for decreasing weight-adjusted lumefantrine dose. Female gender was positively associated. No clear association of C [lum]_day3 _with age was noted. In the model for C [lum]_day7 _(Table 3), unsupervised treatment retained its strong negative effect. Age <5 y also had a strong negative association. A history of dizziness and attendance of the regional referral hospital were both associated with a lower C [lum]_day7_. The trend for weight-adjusted lumefantrine dose was less clear; values between 50 and 79 mg/Kg were associated with lower C [lum]_day7 _but no such association was shown for the under 50 mg/Kg group. Gametocyte carriage at inclusion was positively associated with C [lum]_day7_. No significant interactions between age, lumefantrine dose and arm were found. Overall, however, both models accounted for only a small fraction of the observed variability in lumefantrine concentrations (adjusted R^2 ^values < 0.25).

**Table 2:** Final main effects linear model of the association between baseline explanatory variables and day 3 lumefantrine concentration in ng/mL (456 patients).

**Variable**	**Coefficient**	**95%CI**		**p-value**	**p-value (trend)**
		lower	upper		
		
female gender	791.4	125.2	1493.3	0.019	
unsupervised treatment	-2538.2	-2995.0	-2046.8	<0.0001	
age					
≥15 years	[reference]				0.358
5–14 years	322.5	-657.8	1388.6	0.530	
<5 years	462.3	-478.7	1480.2	0.345	
					
lumefantrine dose (mg/Kg)					
≥80	[reference]				0.033
65–79	-463.2	-1336.2	488.2	0.329	
50–64	-1009.2	-1892.5	-35.4	0.043	
<50	-1237.2	-2386.6	79.5	0.064	
					
history of vomiting at inclusion	-688.6	-1294.1	-44.8	0.036	
constant	5778.1	4313.1	7457.0		
			adjusted R^2^: 0.164		
			p-value for goodness of fit: <0.0001		

**Table 3:** Final main effects linear model of the association between baseline explanatory variables and day 7 lumefantrine concentration in ng/mL (433 patients).

**Variable**	**Coefficient**	**95%CI**		**p-value**	**p-value (trend)**
		lower	upper		
		
referred from hospital OPD (vs. from outlying clinics)	-198.3	-316.9	-63.1	0.005	
unsupervised treatment	-463.3	-536.8	-381.0	<0.0001	
age					
≥15 years	[reference]				<0.0001
5–14 years	-54.6	-233.3	158.4	0.591	
<5 years	-357.9	-483.8	-208.6	<0.0001	
					
lumefantrine dose (mg/Kg)					
≥80	[reference]				0.375
65–79	-168.6	-319.3	9.1	0.062	
50–64	-187.5	-348.4	5.1	0.056	
<50	-36.1	-287.0	286.8	0.806	
					
gametocytaemic at inclusion	395.2	89.5	775.7	0.009	
history of dizziness at inclusion	-284.4	-414.9	-131.1	0.001	
constant					
			adjusted R^2^: 0.219		
			p-value for goodness of fit: <0.0001		

### Re-infection risk

Of 957 patients enrolled in the trial, 946 were ITT-eligible and included in the Cox proportional hazards model. All patients were parasite negative by day 3. By day 28, there were 33 PCR-confirmed re-infections but no parasitological failures. Total person-time of follow up was 26,005 person-days, giving a re-infection rate of 0.00127 (33/26,005) person-days, or 0.46 per person-year. All re-infections occurred on or after day 21, and 22/34 (64.7%) on day 28. After adjusting for age and arm, decreasing weight-adjusted lumefantrine dose was associated significantly with a higher hazard of re-infection, although confidence intervals were wide (Table 4). In the subset of patients with an available C [lum]_day7 _result, the probability of re-infection increased significantly as C [lum]_day7 _decreased, and no re-infections occurred in patients with levels ≥400 ng/mL (Table 5). We could not perform a multivariate regression, because C [lum]_day7 _lies in the causal pathway between the dependent (re-infection) and other explanatory variables. However, stratifying by age group, the only strong confounder detected by the Cox model, also showed a significant risk of re-infection with declining age (Table 5).

**Table 4:** Cox proportional hazards model of relative hazards of re-infection during 28 day follow-up (946 patients).

**Variable**	**Relative hazard**	**95%CI**		**p-value**	**p-value (trend)**
		lower	upper		
			
unsupervised treatment	1.77	0.82	3.79	0.144	
age group					<0.001
15 years and above	1.00 [reference]				
5–14 years	4.05	1.10	14.94	0.036	
<5 years	8.44	2.71	26.27	<0.001	
					
lumefantrine dose (mg/Kg)					0.003
≥80	1.00 [reference]				
65–79	7.50	0.95	59.12	0.056	
50–64	13.95	1.82	106.65	0.011	
<50	12.72	1.08	150.22	0.043	

**Table 5:** Log-rank test of probability of survival (i.e. remaining re-infection free), by age group and day 7 lumefantrine concentration.

**C [lum]**_day7 _(ng/mL)	**< 5 years**			**5–14 years**			**≥15 years**			**Total**		
	n_c_	n_r_	p_surv_	n_c_	n_r_	p_surv_	n_c_	n_r_	p_surv_	n_c_	n_r_	p_surv_
				
<200	47	5	0.889	32	4	0.871	33	2	0.938	112	11	0.898
200–399	51	3	0.940	77	2	0.987	63	2	0.968	191	7	0.968
400–599	20	0	1.000	34	0	1.000	33	0	1.000	87	0	1.000
≥600	11	0	1.000	30	0	1.000	21	0	1.000	62	0	1.000
												
p-value (trend):	0.002			0.283			0.399			<0.001		

n_c _= number in category n_r _= number of re-infections p_surv _= probability of survival

## Discussion

This study has generated new and valuable information on the pharmacokinetics and dose-dependent effects of A/L in malaria patients of all ages at a sub-Saharan African site experiencing stable transmission of multi-drug resistant *P. falciparum*, namely the kind of setting where highly efficacious ACTs like artemether-lumefantrine are needed and will probably be most beneficial.

In these Ugandan patients, we found that both C [lum]_day3 _and C [lum]_day7 _displayed up to fifteen-fold inter-individual variability, consistent with other studies [13]. Median lumefantrine levels were significantly lower in unsupervised patients, but these differences did not affect cure rates at 28 days [8]. The C [lum]_day3 _concentrations in the unsupervised patients were similar to those found in a previous adherence study in Mbarara [4].

C [lum]_day3 _and C [lum]_day7 _correlated well, but were affected differently by certain baseline patient variables. The peak lumefantrine concentration after the three day regimen occurs some 70 hours after intake of the first dose [3,13]. As a close surrogate, we sampled patients on day 3, namely at approximately 72 hours. The C [lum]_day3 _may represent mostly drug intake, absorption from the small bowel and distribution: this could explain why unsupervised treatment, vomiting and a lower weight-adjusted lumefantrine dose were associated with a reduced C [lum]_day3_. By contrast, the C [lum]_day7 _could be the result of drug metabolism and elimination (clearance), principally by the liver and biliary system: children under five had significantly lower C [lum]_day7_, possibly because of enhanced metabolism and clearance. Interestingly, age was not a significant explanatory factor for the C [lum]_day3 _in this study, a contrasting finding with the A/L adherence study [4]. The associations of female gender with C [lum]_day3 _and dizziness at baseline with C [lum]_day7 _are harder to explain, and might be spurious results because of multiple significance tests. In Thai patients, age, weight, and gender were not associated with any of the main PK parameters but the trial did not include children of less than six years of age [3].

Despite highlighting these interesting associations, on the whole our models did not satisfactorily explain the variance in C [lum]. Previous studies have shown that the large effect on lumefantrine PK of lipid intake, which our study was not designed to measure, far outweighs other factors [13].

These findings should be generalized with caution. In Asia, Chinese patients had higher peak lumefantrine on day 3 than Thai patients treated with the same A/L regimen (four tablets over 48 hours) who in turn had higher values than European patients [3,13]. Median C [lum]_day3 _results in our study are broadly consistent with those of Thai patients but lower than those in Chinese patients, even though patients in this study received the six dose regimen. Apart from food intake, pharmacogenetic factors (e.g. the wide variability in CYP 3A, the cytochrome P450 enzyme responsible for artemether and lumefantrine metabolism [3,13]) and differences in nutritional status in African patients may be important and are avenues for further research.

In the multi-drug resistant areas of western Thailand, a day 7 lumefantrine concentration below 280 ng/mL was a reasonable predictor of treatment failure. In this study, there were no treatment failures despite day 7 lumefantrine concentrations <280 ng/ml in 45% of all patients, a finding probably due to the high parasite sensitivity to lumefantrine in Uganda.

By contrast, a day 7 lumefantrine concentration of <400 ng/ml and receiving a lower dose per Kg of lumefantrine were risk factors for re-infection during follow-up; this risk was thirteen-fold higher in patients receiving less than 65 mg/Kg compared to those receiving ≥80 mg/Kg. Most re-infections became visible in the blood 21 to 28 days after start of treatment, when lumefantrine levels would be expected to be very low, and sub-therapeutic. In our study almost 50% (134/286) of children under 5 years received less than 65 mg/Kg of lumefantrine; these children also experienced an additional age-related, eight-fold higher risk of re-infection compared to adults. Parasite re-infection has received far less attention than recrudescence. Nonetheless, in the era of highly efficacious ACTs like A/L, preventing re-infections may become an important public health issue, especially in children under five in stable high transmission areas and individuals during malaria epidemics; in both groups, re-infections cause high malaria morbidity and mortality. Our findings suggest that, in routine practice, low adherence and poor lipid intake could result in re-infection rates that would negate the benefits of high A/L efficacy.

This *in vivo *study extended to only 28 days. Had patients been monitored for 42 days, as recommended by some, we could have detected later recrudescences [14]. The sample of patients may not have been fully representative of a typical outpatient population because the trial had specific entry criteria, and the study environment may have modified patients' adherence patterns. The study was not designed to estimate pharmacokinetic parameters such as peak concentration and terminal half-life. Lumefantrine levels could only be measured on days 3 and 7. Both of these time point measures are crude surrogates of the AUC, the key pharmacokinetic determinant of cure. Further modelling in different settings will be required to determine how useful these markers are.

Despite the limitations of this study, it is clear that a high day 28 A/L cure rate can be achieved despite low plasma lumefantrine levels, even among unsupervised patients. This apparently favourable situation may, however, set up conditions for the selection of resistant parasites, especially in young children who not only harbour high biomass infections but also achieve the lowest day 7 lumefantrine levels. The prevention of *de novo *resistance to ACT combinations is clearly a crucial consideration that probably overrides the desirability of preventing novel infections through a post-treatment prophylactic effect [15]. In this regard, adherence to the six dose A/L regimen, now recommended by WHO, will be critical. Other efficacious ACTs may also be unable to significantly prevent re-infections in high transmission areas, where the simultaneous deployment of an effective ACT and insecticide-impregnated bed nets is needed.

## Conclusion

A/L is a precious tool in the available arsenal against malaria morbidity and mortality, especially in African children. Although it needs to be made widely available to populations in need, care should also be taken to avoid creating favourable conditions for the emergence of lumefantrine-resistant malaria strains [16]. Further research is needed to explore the determinants of sub-optimal lumefantrine plasma levels and how to overcome these. Health systems deploying A/L should develop and strengthen strategies to maximize the effectiveness of the six-dose regimen, for which both high adherence and appropriate food intake are proven imperatives. Monitoring lumefantrine resistance should be an essential element of widespread implementation of this valuable drug combination. New studies should explore the synergistic effects of using ACTs and insecticide-impregnated bed nets.

## Authors' contributions

FC, PP, CF, FB, WRJT and JPG designed the trial. FC and PP analysed data. CF, FB, SB, FG, ER, JB and IK were in charge of trial recruitment and on-site supervision, and contributed to data analysis. JKi, JKy and LF designed and supervised laboratory procedures, with the exception of pharmacokinetic assays, and interpreted PCR data. FC, PP, WRJ and JPG interpreted findings and wrote this paper with the contribution of other authors. All authors read and approved the final manuscript.
